# Acrylato[tris­(1-methyl­benzimidazol-2-ylmeth­yl)amine]zinc(II) perchlorate–dimethyl­formamide–methanol (1/1/1.5) at 153 (2) K

**DOI:** 10.1107/S1600536807068675

**Published:** 2008-01-09

**Authors:** Yongqiang Tian, Huilu Wu, Ruirui Yun, Jingkun Yuan, Jian Ding

**Affiliations:** a School of Chemical and Biological Engineering, Lanzhou Jiaotong University, Lanzhou 730070, People’s Republic of China

## Abstract

In the title complex, [Zn(C_3_H_3_O_2_)(C_27_H_27_N_7_)](ClO_4_)·C_3_H_7_NO·1.5CH_4_O, the Zn^II^ ion is five-coordinated by four N atoms from a tris­(1-methyl­benzimidazol-2-ylmeth­yl)amine (Mentb) ligand and one O atom from an acrylate ligand in a distorted trigonal–bipyramidal geometry with approximate mol­ecular *C*_3_ symmetry. The atoms of the acrylate ligand are disordered over two sites, with approximate occupancies of 0.84 and 0.16. In addition, a methanol solvent mol­ecule is disordered over two sites with equal occupancies. In the crystal structure, the full-occupancy methanol is linked to a dimethyl­formamide mol­ecule by an inter­molecular O—H⋯O hydrogen bond.

## Related literature

For related literature, see: Youngme *et al.* (2007 ▶). For bond-length data, see: Allen *et al.* (1987 ▶).
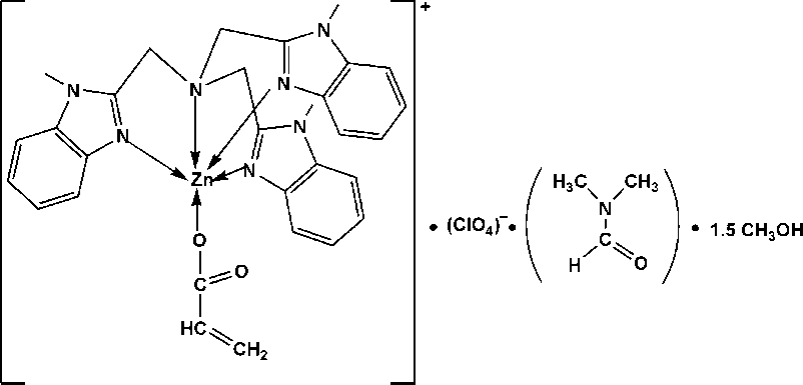


## Experimental

### 

#### Crystal data

[Zn(C_3_H_3_O_2_)(C_27_H_27_N_7_)](ClO_4_)·C_3_H_7_NO·1.5CH_4_O
*M* *_r_* = 806.61
Triclinic, 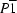

*a* = 11.3766 (4) Å
*b* = 13.9606 (4) Å
*c* = 14.4355 (5) Å
α = 108.579 (1)°
β = 111.011 (1)°
γ = 100.075 (1)°
*V* = 1917.33 (11) Å^3^
*Z* = 2
Mo *K*α radiation
μ = 0.77 mm^−1^
*T* = 153 (2) K
0.59 × 0.56 × 0.40 mm

#### Data collection

Rigaku R-AXIS SPIDER diffractometer
Absorption correction: multi-scan (Higashi; 1995 ▶) *T*_min_ = 0.659, *T*_max_ = 0.748
18907 measured reflections
8711 independent reflections
7735 reflections with *I* > 2σ(*I*)
*R*_int_ = 0.024

#### Refinement

*R*[*F* ^2^ > 2σ(*F* ^2^)] = 0.045
*wR*(*F* ^2^) = 0.144
*S* = 1.05
8711 reflections
523 parameters
28 restraints
H-atom parameters constrained
Δρ_max_ = 1.29 e Å^−3^
Δρ_min_ = −0.73 e Å^−3^

### 

Data collection: *RAPID-AUTO* (Rigaku/MSC 2004 ▶); cell refinement: *RAPID-AUTO*; data reduction: *RAPID-AUTO*; program(s) used to solve structure: *SHELXS97* (Sheldrick, 2008 ▶); program(s) used to refine structure: *SHELXL97* (Sheldrick, 2008 ▶); molecular graphics: *SHELXTL* (Bruker, 1997 ▶) and *PLATON* (Spek, 2003 ▶); software used to prepare material for publication: *SHELXTL*.

## Supplementary Material

Crystal structure: contains datablocks global, I. DOI: 10.1107/S1600536807068675/lh2580sup1.cif

Structure factors: contains datablocks I. DOI: 10.1107/S1600536807068675/lh2580Isup2.hkl

Additional supplementary materials:  crystallographic information; 3D view; checkCIF report

## Figures and Tables

**Table 1:** Selected bond lengths (Å)

Zn—O1	1.988 (2)
Zn—N3	2.0433 (19)
Zn—N1	2.0564 (19)
Zn—N5	2.071 (2)
Zn—N7	2.4497 (19)

**Table 2:** Hydrogen-bond geometry (Å, °)

*D*—H⋯*A*	*D*—H	H⋯*A*	*D*⋯*A*	*D*—H⋯*A*
O8—H8*O*⋯O7^i^	0.84	1.93	2.768 (3)	180

Symmetry code: (i) 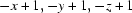.

**Fig. 1.**
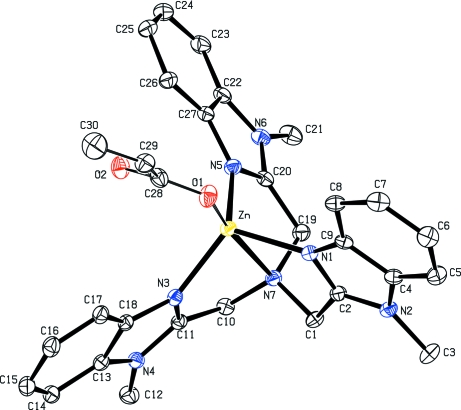
The molecular structure of the cation of the title compound. Hydrogen atoms have been omitted and the displacement ellipsoids are shown at the 30% probability level. The disorder is not shown.

**Fig. 2.**
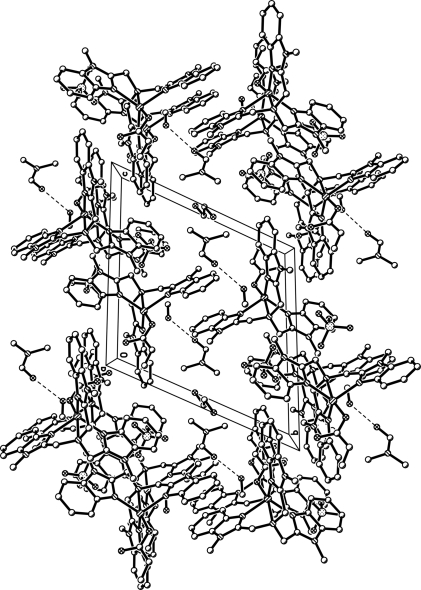
The Packing of the title compound. H atoms are omitted for clarity. The donor-acceptor distances of hydrogen bonds are shown as dashed lines. The disorder is not shown.

## supplementary crystallographic information

### Comment

The asymmetric unit of the title compound consists of a discrete [Zn(Mentb)(acrylate)] cation (Fig. 1), a perchlorate anion, a DMF molecule and 1.5 molecules of methanol. The zinc ion is five-coordinated with a N_4_O ligand set. The Mentb ligand acts as a tetradentate N-donor, and an O atom of a carboxylate group of the acrylate ligand completes the coordination. The coordination geometry of the Zn^II^ may be best described as distorted trigonal bipyramid (τ = 0.85), with approximate site symmetry C_3_. The parameter τ is defined as (β - α)/60 [where β = O1—Zn—N7, α = O1—Zn—N5] and its value varies from 0 (in regular square-based pyramidal) to 1 (in regular trigonal bipyramidal) [Youngme *et al.*, 2007]. This geometry is assumed by the Zn^II^ ion to relieve the steric crowding. The equatorial plane is occupied by three N atoms of three benzimidazolyl groups, while the Zn^II^ ion protrudes towards O1 by 0.558 (8) Å from the plane of atoms N1/N3/N5. The axial positions are occupyied by N7 and O1. The three benzimidazole ring arms of the Mentb ligand form a cone-shaped cavity. The distance between Zn^II^ and O2 is 3.068 (2) Å, so O2 is not considered coordinated. The distances within the ligands are normal [Allen *et al.*, 1987]. The crystal structure is stabilized by weak intermolecular O—H···O hydrogen bonds and weak π···π stacking interactions with *Cg*1···*Cg*2^i^ = 3.465 (2)Å [symmetry code: (i) 1 - *x*, 1 - *y*, 1 - *z*] and a perpendicular distance of 3.437 Å, where *Cg*1 and *Cg*2 are the centroids defined by atoms C4—C9 and N1/C2/N2/C4/C9 respectively.

### Experimental

To a stired solution of tris(*N*-methylbenzimidazol-2-ylmethyl)amine (0.0899 g, 0.2 mmol) in hot MeOH (10 ml) was added Zn(ClO_4_)_2_ (H_2_O)_6_ (0.0745 g, 0.2 mmol), followed by a solution of Na(acrylate) (0.0188 g, 0.2 mmol) in MeOH (5 ml). A colorless crystalline product formed rapidly. The precipitate was filtered off, washed with MeOH and absolute Et_2_O, and dried *in vacuo*. The dried precipitate was dissolved in DMF to form a colorless solution that was allowed to evaporate at room temperature. Colorless crystals suitable for X-ray diffraction studies were obtained after two weeks. Yield, 0.092 g (57%). (found: C, 51.20; H, 5.08; N,13.76. Calcd. for C34.50H43ClN8O8.50Zn: C, 51.37; H, 5.37; N, 13.89)

### Refinement

The atoms of the acrylate ligand are disordered over two sites with refined occupancies of 0.836 (5) and 0.164 (5) for the minimum and maximum components, respectively. The 0.5 occupancy methanol molecule is disordered over two sites with equal occupancies. All H atoms were found in difference electron maps and were subsequently refined in a riding-model approximation with C—H distances ranging from 0.95 to 0.99 Å and O—H distance 0.84 Å and *U*_iso_(H) = 1.2 *U*_eq_(C) or 1.5 *U*_eq_(C_methyl_ or O).

### Crystal data

**Table:**

[Zn(C_3_H_3_O_2_)(C_27_H_27_N_7_)](ClO_4_)·C_3_H_7_NO·1.5CH_4_O	*Z* = 2
*M**_r_* = 806.61	*F*_000_ = 842
Triclinic, *P*1	*D*_x_ = 1.397 Mg m^−^^3^
Hall symbol: -P 1	Mo *K*α radiation λ = 0.71073 Å
*a* = 11.3766 (4) Å	Cell parameters from 16325 reflections
*b* = 13.9606 (4) Å	θ = 3.0–27.5º
*c* = 14.4355 (5) Å	µ = 0.77 mm^−^^1^
α = 108.579 (1)º	*T* = 153 (2) K
β = 111.011 (1)º	Block, colorless
γ = 100.075 (1)º	0.59 × 0.56 × 0.40 mm
*V* = 1917.33 (11) Å^3^	

### Data collection

**Table:**

Rigaku R-axis SPIDER diffractometer	8711 independent reflections
Radiation source: Rotating Anode	7735 reflections with *I* > 2σ(*I*)
Monochromator: graphite	*R*_int_ = 0.024
*T* = 153(2) K	θ_max_ = 27.5º
ω scans	θ_min_ = 3.0º
Absorption correction: multi-scan(Higashi; 1995)	*h* = −14→14
*T*_min_ = 0.659, *T*_max_ = 0.748	*k* = −18→17
18907 measured reflections	*l* = −18→18

### Refinement

**Table:**

Refinement on *F*^2^	Secondary atom site location: difference Fourier map
Least-squares matrix: full	Hydrogen site location: inferred from neighbouring sites
*R*[*F*^2^ > 2σ(*F*^2^)] = 0.045	H-atom parameters constrained
*wR*(*F*^2^) = 0.144	*w* = 1/[σ^2^(*F*_o_^2^) + (0.088*P*)^2^ + 1.5221*P*] where *P* = (*F*_o_^2^ + 2*F*_c_^2^)/3
*S* = 1.05	(Δ/σ)_max_ = 0.001
8711 reflections	Δρ_max_ = 1.29 e Å^−^^3^
523 parameters	Δρ_min_ = −0.73 e Å^−^^3^
28 restraints	Extinction correction: none
Primary atom site location: structure-invariant direct methods	

### Special details

**Table:**

Geometry. All e.s.d.'s (except the e.s.d. in the dihedral angle between two l.s. planes) are estimated using the full covariance matrix. The cell e.s.d.'s are taken into account individually in the estimation of e.s.d.'s in distances, angles and torsion angles; correlations between e.s.d.'s in cell parameters are only used when they are defined by crystal symmetry. An approximate (isotropic) treatment of cell e.s.d.'s is used for estimating e.s.d.'s involving l.s. planes.
Refinement. Refinement of *F*^2^ against ALL reflections. The weighted *R*-factor *wR* and goodness of fit *S* are based on *F*^2^, conventional *R*-factors *R* are based on *F*, with *F* set to zero for negative *F*^2^. The threshold expression of *F*^2^ > σ(*F*^2^) is used only for calculating *R*-factors(gt) *etc*. and is not relevant to the choice of reflections for refinement. *R*-factors based on *F*^2^ are statistically about twice as large as those based on *F*, and *R*- factors based on ALL data will be even larger.

### Fractional atomic coordinates and isotropic or equivalent isotropic displacement parameters (Å^2^)

**Table:**

	*x*	*y*	*z*	*U*_iso_*/*U*_eq_	Occ. (<1)
Zn	0.35354 (2)	0.15960 (2)	0.32037 (2)	0.02210 (10)	
N1	0.43780 (19)	0.29536 (15)	0.46350 (16)	0.0226 (4)	
N2	0.4269 (2)	0.41504 (16)	0.60253 (17)	0.0259 (4)	
N3	0.16855 (19)	0.14640 (15)	0.21459 (15)	0.0230 (4)	
N4	−0.05296 (19)	0.08379 (15)	0.14418 (16)	0.0249 (4)	
N5	0.3625 (2)	0.02424 (16)	0.34902 (16)	0.0250 (4)	
N6	0.3172 (2)	−0.08938 (17)	0.42042 (17)	0.0288 (4)	
N7	0.21188 (19)	0.14020 (15)	0.41159 (16)	0.0233 (4)	
C1	0.2265 (2)	0.24986 (19)	0.4776 (2)	0.0262 (5)	
H1A	0.2102	0.2524	0.5411	0.031*	
H1B	0.1609	0.2751	0.4335	0.031*	
C2	0.3647 (2)	0.31989 (18)	0.51553 (19)	0.0235 (4)	
C3	0.3742 (3)	0.4680 (2)	0.6758 (2)	0.0363 (6)	
H3A	0.2768	0.4443	0.6369	0.054*	
H3B	0.4098	0.5456	0.7010	0.054*	
H3C	0.4007	0.4496	0.7387	0.054*	
C4	0.5506 (2)	0.45659 (19)	0.6080 (2)	0.0258 (5)	
C5	0.6552 (3)	0.5515 (2)	0.6807 (2)	0.0337 (5)	
H5A	0.6503	0.6026	0.7400	0.040*	
C6	0.7666 (3)	0.5675 (2)	0.6621 (2)	0.0371 (6)	
H6A	0.8405	0.6309	0.7103	0.045*	
C7	0.7735 (3)	0.4929 (2)	0.5744 (2)	0.0357 (6)	
H7A	0.8516	0.5072	0.5641	0.043*	
C8	0.6690 (2)	0.3983 (2)	0.5020 (2)	0.0282 (5)	
H8A	0.6740	0.3475	0.4426	0.034*	
C9	0.5564 (2)	0.38108 (18)	0.52022 (19)	0.0238 (4)	
C10	0.0769 (2)	0.07969 (19)	0.32346 (19)	0.0254 (5)	
H10A	0.0104	0.0998	0.3480	0.030*	
H10B	0.0603	0.0021	0.3028	0.030*	
C11	0.0646 (2)	0.10465 (17)	0.22763 (19)	0.0230 (4)	
C12	−0.1853 (2)	0.0394 (2)	0.1352 (2)	0.0323 (5)	
H12A	−0.1830	−0.0124	0.1678	0.048*	
H12B	−0.2502	0.0036	0.0581	0.048*	
H12C	−0.2111	0.0973	0.1735	0.048*	
C13	−0.0252 (2)	0.11589 (17)	0.07074 (19)	0.0248 (4)	
C14	−0.1086 (3)	0.1126 (2)	−0.0289 (2)	0.0303 (5)	
H14A	−0.2028	0.0845	−0.0587	0.036*	
C15	−0.0471 (3)	0.1523 (2)	−0.0823 (2)	0.0338 (5)	
H15A	−0.1005	0.1523	−0.1501	0.041*	
C16	0.0931 (3)	0.1928 (2)	−0.0383 (2)	0.0335 (5)	
H16A	0.1318	0.2195	−0.0772	0.040*	
C17	0.1751 (3)	0.19458 (19)	0.06038 (19)	0.0279 (5)	
H17A	0.2693	0.2217	0.0897	0.033*	
C18	0.1145 (2)	0.15516 (18)	0.11509 (18)	0.0241 (4)	
C19	0.2620 (2)	0.0809 (2)	0.4757 (2)	0.0274 (5)	
H19A	0.1895	0.0409	0.4852	0.033*	
H19B	0.3340	0.1307	0.5487	0.033*	
C20	0.3142 (2)	0.0051 (2)	0.41521 (19)	0.0259 (5)	
C21	0.2753 (3)	−0.1350 (2)	0.4862 (2)	0.0375 (6)	
H21A	0.2256	−0.0941	0.5159	0.056*	
H21B	0.3540	−0.1319	0.5462	0.056*	
H21C	0.2182	−0.2097	0.4405	0.056*	
C22	0.3701 (2)	−0.1366 (2)	0.3518 (2)	0.0290 (5)	
C23	0.3908 (3)	−0.2346 (2)	0.3233 (2)	0.0381 (6)	
H23A	0.3668	−0.2842	0.3510	0.046*	
C24	0.4480 (3)	−0.2564 (2)	0.2530 (2)	0.0438 (7)	
H24A	0.4641	−0.3227	0.2319	0.053*	
C25	0.4830 (3)	−0.1842 (2)	0.2118 (2)	0.0417 (7)	
H25A	0.5244	−0.2015	0.1650	0.050*	
C26	0.4583 (3)	−0.0871 (2)	0.2381 (2)	0.0332 (5)	
H26A	0.4804	−0.0385	0.2087	0.040*	
C27	0.4004 (2)	−0.06388 (19)	0.30861 (19)	0.0266 (5)	
C28	0.4818 (2)	0.1641 (2)	0.18395 (19)	0.0314 (5)	
O1	0.4880 (2)	0.21199 (19)	0.27462 (17)	0.0352 (6)	0.836 (5)
O2	0.4042 (2)	0.06708 (18)	0.12040 (18)	0.0360 (6)	0.836 (5)
C29	0.5724 (3)	0.2162 (3)	0.1495 (3)	0.0322 (7)	0.836 (5)
H29	0.6360	0.2843	0.2008	0.039*	0.836 (5)
C30	0.5724 (4)	0.1770 (3)	0.0555 (3)	0.0584 (9)	
H30A	0.5104	0.1092	0.0019	0.070*	0.836 (5)
H30B	0.6344	0.2161	0.0400	0.070*	0.836 (5)
O1'	0.4269 (12)	0.1035 (7)	0.2132 (10)	0.052 (4)	0.164 (5)
O2'	0.4857 (14)	0.2630 (5)	0.2149 (10)	0.061 (5)	0.164 (5)
C29'	0.5255 (19)	0.1223 (7)	0.0992 (12)	0.070 (9)	0.164 (5)
H29'	0.5187	0.0489	0.0745	0.084*	0.164 (5)
H30C	0.5809	0.2507	0.0778	0.070*	0.164 (5)
H30D	0.5983	0.1436	0.0011	0.070*	0.164 (5)
O9	−0.3164 (15)	−0.4481 (12)	−0.0180 (13)	0.097 (4)	0.25
H9A	−0.3562	−0.4937	−0.0841	0.145*	0.25
C35	−0.404 (3)	−0.498 (2)	0.020 (2)	0.108 (6)	0.25
H35A	−0.3769	−0.4534	0.0966	0.161*	0.25
H35B	−0.3972	−0.5691	0.0121	0.161*	0.25
H35C	−0.4964	−0.5054	−0.0242	0.161*	0.25
O9'	−0.4896 (14)	−0.5339 (13)	−0.0429 (14)	0.098 (4)	0.25
H9'A	−0.5219	−0.5595	−0.1120	0.147*	0.25
C35'	−0.3528 (2)	−0.45924 (15)	0.00898 (18)	0.108 (6)	0.25
H35D	−0.3532	−0.3997	−0.0130	0.161*	0.25
H35E	−0.2961	−0.4968	−0.0140	0.161*	0.25
H35F	−0.3180	−0.4316	0.0884	0.161*	0.25
O7	1.1335 (2)	0.55543 (15)	0.72271 (18)	0.0648 (7)	
N10	1.0423 (2)	0.51106 (15)	0.82520 (18)	0.0563 (7)	
C31	0.9669 (2)	0.42510 (15)	0.84068 (18)	0.100 (2)	
H31A	0.9486	0.3558	0.7833	0.150*	
H31B	0.8827	0.4357	0.8368	0.150*	
H31C	1.0195	0.4268	0.9122	0.150*	
C32	1.0725 (5)	0.6191 (3)	0.8999 (4)	0.0653 (10)	
H32A	1.1206	0.6690	0.8806	0.098*	
H32B	1.1281	0.6291	0.9743	0.098*	
H32C	0.9895	0.6328	0.8955	0.098*	
C33	1.0770 (4)	0.4894 (3)	0.7448 (3)	0.0534 (8)	
H33A	1.0563	0.4162	0.7003	0.064*	
O8	−0.0521 (2)	0.28742 (17)	0.33417 (18)	0.0412 (5)	
H8O	−0.0767	0.3351	0.3169	0.062*	
C34	−0.1037 (4)	0.2828 (4)	0.4076 (4)	0.0650 (11)	
H34A	−0.0733	0.2337	0.4388	0.097*	
H34B	−0.2013	0.2572	0.3693	0.097*	
H34C	−0.0728	0.3543	0.4661	0.097*	
Cl	−0.10330 (7)	−0.17834 (5)	0.32322 (6)	0.03704 (16)	
O3	−0.1742 (3)	−0.1134 (3)	0.2867 (3)	0.0787 (10)	
O4	−0.1929 (3)	−0.2823 (2)	0.2867 (2)	0.0705 (8)	
O5	−0.0381 (2)	−0.1306 (2)	0.44072 (19)	0.0566 (6)	
O6	−0.0026 (2)	−0.1836 (2)	0.28517 (18)	0.0489 (5)	

### Atomic displacement parameters (Å^2^)

**Table:**

	*U*^11^	*U*^22^	*U*^33^	*U*^12^	*U*^13^	*U*^23^
Zn	0.02388 (15)	0.02273 (15)	0.02353 (15)	0.00978 (10)	0.01317 (11)	0.01000 (11)
N1	0.0230 (9)	0.0219 (9)	0.0252 (9)	0.0086 (7)	0.0122 (8)	0.0103 (8)
N2	0.0308 (10)	0.0231 (10)	0.0286 (10)	0.0124 (8)	0.0160 (8)	0.0113 (8)
N3	0.0266 (9)	0.0198 (9)	0.0219 (9)	0.0067 (7)	0.0115 (8)	0.0076 (7)
N4	0.0239 (9)	0.0212 (9)	0.0255 (9)	0.0068 (7)	0.0087 (8)	0.0076 (8)
N5	0.0283 (10)	0.0239 (9)	0.0265 (9)	0.0112 (8)	0.0136 (8)	0.0117 (8)
N6	0.0297 (10)	0.0309 (11)	0.0319 (10)	0.0132 (8)	0.0130 (9)	0.0192 (9)
N7	0.0231 (9)	0.0233 (9)	0.0239 (9)	0.0083 (7)	0.0110 (8)	0.0094 (8)
C1	0.0280 (11)	0.0257 (11)	0.0296 (11)	0.0105 (9)	0.0179 (10)	0.0101 (9)
C2	0.0253 (11)	0.0244 (11)	0.0248 (10)	0.0111 (9)	0.0127 (9)	0.0114 (9)
C3	0.0470 (15)	0.0295 (13)	0.0374 (14)	0.0153 (11)	0.0273 (13)	0.0086 (11)
C4	0.0283 (11)	0.0226 (11)	0.0312 (12)	0.0109 (9)	0.0137 (10)	0.0151 (9)
C5	0.0409 (14)	0.0198 (11)	0.0373 (13)	0.0093 (10)	0.0159 (12)	0.0102 (10)
C6	0.0340 (13)	0.0223 (12)	0.0452 (15)	0.0015 (10)	0.0127 (12)	0.0120 (11)
C7	0.0305 (13)	0.0283 (13)	0.0507 (16)	0.0069 (10)	0.0186 (12)	0.0201 (12)
C8	0.0277 (11)	0.0246 (11)	0.0380 (13)	0.0109 (9)	0.0165 (10)	0.0163 (10)
C9	0.0249 (11)	0.0217 (11)	0.0297 (11)	0.0107 (8)	0.0115 (9)	0.0158 (9)
C10	0.0232 (11)	0.0256 (11)	0.0281 (11)	0.0068 (9)	0.0118 (9)	0.0123 (9)
C11	0.0246 (11)	0.0177 (10)	0.0249 (10)	0.0073 (8)	0.0105 (9)	0.0070 (8)
C12	0.0242 (11)	0.0331 (13)	0.0358 (13)	0.0075 (9)	0.0120 (10)	0.0125 (11)
C13	0.0282 (11)	0.0161 (10)	0.0258 (11)	0.0067 (8)	0.0106 (9)	0.0054 (8)
C14	0.0316 (12)	0.0240 (11)	0.0268 (11)	0.0098 (9)	0.0072 (10)	0.0066 (9)
C15	0.0433 (14)	0.0298 (13)	0.0242 (11)	0.0138 (11)	0.0109 (11)	0.0098 (10)
C16	0.0443 (15)	0.0304 (13)	0.0280 (12)	0.0122 (11)	0.0178 (11)	0.0127 (10)
C17	0.0338 (12)	0.0235 (11)	0.0245 (11)	0.0090 (9)	0.0127 (10)	0.0084 (9)
C18	0.0288 (11)	0.0188 (10)	0.0218 (10)	0.0086 (8)	0.0096 (9)	0.0062 (8)
C19	0.0314 (12)	0.0311 (12)	0.0270 (11)	0.0139 (10)	0.0164 (10)	0.0151 (10)
C20	0.0243 (11)	0.0298 (12)	0.0258 (11)	0.0112 (9)	0.0092 (9)	0.0152 (9)
C21	0.0387 (14)	0.0441 (15)	0.0445 (15)	0.0166 (12)	0.0200 (12)	0.0328 (13)
C22	0.0271 (12)	0.0303 (12)	0.0280 (12)	0.0129 (9)	0.0072 (10)	0.0143 (10)
C23	0.0460 (15)	0.0334 (14)	0.0358 (14)	0.0202 (12)	0.0112 (12)	0.0195 (11)
C24	0.0578 (18)	0.0359 (15)	0.0364 (14)	0.0306 (14)	0.0127 (14)	0.0147 (12)
C25	0.0546 (18)	0.0435 (16)	0.0324 (13)	0.0323 (14)	0.0191 (13)	0.0140 (12)
C26	0.0417 (14)	0.0348 (14)	0.0301 (12)	0.0221 (11)	0.0176 (11)	0.0149 (11)
C27	0.0268 (11)	0.0266 (11)	0.0253 (11)	0.0128 (9)	0.0081 (9)	0.0113 (9)
C28	0.0234 (11)	0.0411 (15)	0.0286 (12)	0.0079 (10)	0.0108 (10)	0.0153 (11)
O1	0.0306 (11)	0.0413 (14)	0.0298 (11)	0.0043 (9)	0.0180 (9)	0.0088 (10)
O2	0.0367 (12)	0.0289 (11)	0.0314 (12)	0.0018 (9)	0.0102 (10)	0.0095 (9)
C29	0.0296 (15)	0.0387 (17)	0.0360 (16)	0.0113 (13)	0.0201 (13)	0.0182 (14)
C30	0.060 (2)	0.085 (3)	0.054 (2)	0.0269 (19)	0.0396 (18)	0.040 (2)
C28'	0.0234 (11)	0.0411 (15)	0.0286 (12)	0.0079 (10)	0.0108 (10)	0.0153 (11)
O1'	0.051 (8)	0.093 (12)	0.061 (9)	0.044 (8)	0.047 (7)	0.057 (9)
O2'	0.046 (8)	0.079 (12)	0.044 (8)	−0.008 (7)	0.017 (7)	0.026 (8)
C29'	0.044 (12)	0.14 (3)	0.070 (16)	0.049 (15)	0.035 (12)	0.079 (19)
C30'	0.060 (2)	0.085 (3)	0.054 (2)	0.0269 (19)	0.0396 (18)	0.040 (2)
O9	0.106 (6)	0.085 (6)	0.089 (6)	0.002 (4)	0.036 (4)	0.048 (5)
C35	0.105 (7)	0.116 (8)	0.096 (7)	0.020 (5)	0.058 (5)	0.030 (5)
O9'	0.083 (6)	0.114 (6)	0.095 (6)	0.038 (4)	0.039 (4)	0.039 (4)
C35'	0.105 (7)	0.116 (8)	0.096 (7)	0.020 (5)	0.058 (5)	0.030 (5)
O7	0.0665 (16)	0.0675 (17)	0.101 (2)	0.0383 (14)	0.0541 (16)	0.0547 (17)
N10	0.0695 (19)	0.0356 (14)	0.0688 (19)	0.0159 (13)	0.0407 (17)	0.0167 (13)
C31	0.152 (5)	0.049 (2)	0.122 (4)	0.015 (3)	0.102 (4)	0.024 (3)
C32	0.078 (3)	0.0409 (19)	0.069 (2)	0.0217 (18)	0.034 (2)	0.0109 (17)
C33	0.062 (2)	0.0419 (17)	0.068 (2)	0.0253 (15)	0.0372 (19)	0.0233 (16)
O8	0.0394 (11)	0.0405 (11)	0.0539 (12)	0.0173 (9)	0.0283 (10)	0.0206 (10)
C34	0.069 (2)	0.093 (3)	0.080 (3)	0.052 (2)	0.055 (2)	0.052 (2)
Cl	0.0412 (4)	0.0373 (3)	0.0420 (4)	0.0115 (3)	0.0224 (3)	0.0240 (3)
O3	0.0457 (14)	0.100 (2)	0.134 (3)	0.0352 (15)	0.0412 (17)	0.092 (2)
O4	0.092 (2)	0.0420 (14)	0.0653 (17)	−0.0064 (13)	0.0423 (16)	0.0146 (12)
O5	0.0525 (14)	0.0726 (17)	0.0428 (12)	0.0167 (12)	0.0285 (11)	0.0151 (12)
O6	0.0566 (13)	0.0657 (15)	0.0430 (11)	0.0255 (11)	0.0333 (11)	0.0290 (11)

### Geometric parameters (Å, °)

**Table:**

Zn—O1	1.988 (2)	C19—C20	1.495 (3)
Zn—O1'	2.021 (7)	C19—H19A	0.9900
Zn—N3	2.0433 (19)	C19—H19B	0.9900
Zn—N1	2.0564 (19)	C21—H21A	0.9800
Zn—N5	2.071 (2)	C21—H21B	0.9800
Zn—N7	2.4497 (19)	C21—H21C	0.9800
N1—C2	1.326 (3)	C22—C23	1.386 (4)
N1—C9	1.393 (3)	C22—C27	1.405 (3)
N2—C2	1.347 (3)	C23—C24	1.376 (4)
N2—C4	1.389 (3)	C23—H23A	0.9500
N2—C3	1.462 (3)	C24—C25	1.392 (5)
N3—C11	1.328 (3)	C24—H24A	0.9500
N3—C18	1.400 (3)	C25—C26	1.394 (4)
N4—C11	1.345 (3)	C25—H25A	0.9500
N4—C13	1.385 (3)	C26—C27	1.386 (4)
N4—C12	1.466 (3)	C26—H26A	0.9500
N5—C20	1.332 (3)	C28—O1	1.235 (3)
N5—C27	1.396 (3)	C28—O2	1.290 (3)
N6—C20	1.351 (3)	C28—C29	1.474 (4)
N6—C22	1.388 (3)	C29—C30	1.292 (5)
N6—C21	1.463 (3)	C29—H29	0.9500
N7—C19	1.464 (3)	C30—H30A	0.9500
N7—C1	1.465 (3)	C30—H30B	0.9500
N7—C10	1.466 (3)	C29'—H29'	0.9500
C1—C2	1.493 (3)	O9—C35	1.473 (15)
C1—H1A	0.9900	O9—H9A	0.8500
C1—H1B	0.9900	C35—H35A	0.9800
C3—H3A	0.9800	C35—H35B	0.9800
C3—H3B	0.9800	C35—H35C	0.9800
C3—H3C	0.9800	O9'—O9'^i^	1.42 (3)
C4—C5	1.393 (3)	O9'—C35'	1.473 (15)
C4—C9	1.398 (3)	O9'—H9'A	0.8500
C5—C6	1.382 (4)	C35'—H9A	1.2583
C5—H5A	0.9500	C35'—H35D	0.9800
C6—C7	1.398 (4)	C35'—H35E	0.9800
C6—H6A	0.9500	C35'—H35F	0.9800
C7—C8	1.389 (4)	O7—C33	1.221 (4)
C7—H7A	0.9500	N10—C33	1.320 (4)
C8—C9	1.393 (3)	N10—C32	1.439 (4)
C8—H8A	0.9500	N10—C31	1.4743
C10—C11	1.497 (3)	C31—H31A	0.9800
C10—H10A	0.9900	C31—H31B	0.9800
C10—H10B	0.9900	C31—H31C	0.9800
C12—H12A	0.9800	C32—H32A	0.9800
C12—H12B	0.9800	C32—H32B	0.9800
C12—H12C	0.9800	C32—H32C	0.9800
C13—C14	1.391 (3)	C33—H33A	0.9500
C13—C18	1.406 (3)	O8—C34	1.396 (4)
C14—C15	1.383 (4)	O8—H8O	0.8399
C14—H14A	0.9500	C34—H34A	0.9800
C15—C16	1.413 (4)	C34—H34B	0.9800
C15—H15A	0.9500	C34—H34C	0.9800
C16—C17	1.385 (4)	Cl—O3	1.417 (3)
C16—H16A	0.9500	Cl—O4	1.426 (2)
C17—C18	1.391 (3)	Cl—O6	1.440 (2)
C17—H17A	0.9500	Cl—O5	1.441 (2)
			
O1—Zn—O1'	40.2 (3)	C16—C17—H17A	121.2
O1—Zn—N3	109.73 (9)	C18—C17—H17A	121.2
O1'—Zn—N3	99.7 (4)	C17—C18—N3	131.3 (2)
O1—Zn—N1	91.83 (8)	C17—C18—C13	120.4 (2)
O1'—Zn—N1	129.8 (3)	N3—C18—C13	108.3 (2)
N3—Zn—N1	113.36 (7)	N7—C19—C20	107.57 (19)
O1—Zn—N5	114.45 (9)	N7—C19—H19A	110.2
O1'—Zn—N5	85.6 (2)	C20—C19—H19A	110.2
N3—Zn—N5	113.95 (8)	N7—C19—H19B	110.2
N1—Zn—N5	111.62 (8)	C20—C19—H19B	110.2
O1—Zn—N7	165.85 (8)	H19A—C19—H19B	108.5
O1'—Zn—N7	153.8 (2)	N5—C20—N6	112.6 (2)
N3—Zn—N7	74.52 (7)	N5—C20—C19	122.9 (2)
N1—Zn—N7	74.26 (7)	N6—C20—C19	124.6 (2)
N5—Zn—N7	74.16 (7)	N6—C21—H21A	109.5
C2—N1—C9	105.68 (19)	N6—C21—H21B	109.5
C2—N1—Zn	118.50 (15)	H21A—C21—H21B	109.5
C9—N1—Zn	135.45 (16)	N6—C21—H21C	109.5
C2—N2—C4	107.03 (19)	H21A—C21—H21C	109.5
C2—N2—C3	127.3 (2)	H21B—C21—H21C	109.5
C4—N2—C3	125.6 (2)	C23—C22—N6	131.5 (2)
C11—N3—C18	105.37 (19)	C23—C22—C27	122.5 (3)
C11—N3—Zn	118.12 (15)	N6—C22—C27	105.9 (2)
C18—N3—Zn	135.63 (16)	C24—C23—C22	116.6 (3)
C11—N4—C13	107.08 (19)	C24—C23—H23A	121.7
C11—N4—C12	126.3 (2)	C22—C23—H23A	121.7
C13—N4—C12	126.6 (2)	C23—C24—C25	122.0 (3)
C20—N5—C27	105.7 (2)	C23—C24—H24A	119.0
C20—N5—Zn	118.67 (16)	C25—C24—H24A	119.0
C27—N5—Zn	135.45 (16)	C24—C25—C26	121.2 (3)
C20—N6—C22	107.2 (2)	C24—C25—H25A	119.4
C20—N6—C21	127.6 (2)	C26—C25—H25A	119.4
C22—N6—C21	125.2 (2)	C27—C26—C25	117.5 (3)
C19—N7—C1	113.95 (19)	C27—C26—H26A	121.2
C19—N7—C10	112.14 (19)	C25—C26—H26A	121.2
C1—N7—C10	113.24 (18)	C26—C27—N5	131.4 (2)
C19—N7—Zn	106.35 (13)	C26—C27—C22	120.0 (2)
C1—N7—Zn	104.88 (13)	N5—C27—C22	108.6 (2)
C10—N7—Zn	105.36 (13)	O1—C28—O2	122.3 (2)
N7—C1—C2	108.60 (18)	O1—C28—C29	119.2 (2)
N7—C1—H1A	110.0	O2—C28—C29	118.3 (2)
C2—C1—H1A	110.0	C28—O1—Zn	122.54 (18)
N7—C1—H1B	110.0	C30—C29—C28	125.5 (3)
C2—C1—H1B	110.0	C30—C29—H29	117.3
H1A—C1—H1B	108.4	C28—C29—H29	117.3
N1—C2—N2	112.7 (2)	C29—C30—H30A	120.0
N1—C2—C1	122.6 (2)	C29—C30—H30B	120.0
N2—C2—C1	124.7 (2)	H30A—C30—H30B	120.0
N2—C3—H3A	109.5	C35—O9—H9A	96.7
N2—C3—H3B	109.5	O9—C35—H35A	109.5
H3A—C3—H3B	109.5	O9—C35—H35B	109.5
N2—C3—H3C	109.5	H35A—C35—H35B	109.5
H3A—C3—H3C	109.5	O9—C35—H35C	109.5
H3B—C3—H3C	109.5	H35A—C35—H35C	109.5
N2—C4—C5	131.8 (2)	H35B—C35—H35C	109.5
N2—C4—C9	105.9 (2)	O9'^i^—O9'—C35'	88.2 (14)
C5—C4—C9	122.3 (2)	O9'^i^—O9'—H9'A	139.6
C6—C5—C4	116.5 (2)	C35'—O9'—H9'A	111.3
C6—C5—H5A	121.8	O9'—C35'—H9A	86.6
C4—C5—H5A	121.8	O9'—C35'—H35D	109.5
C5—C6—C7	121.8 (2)	H9A—C35'—H35D	74.3
C5—C6—H6A	119.1	O9'—C35'—H35E	109.5
C7—C6—H6A	119.1	H9A—C35'—H35E	52.4
C8—C7—C6	121.6 (2)	H35D—C35'—H35E	109.5
C8—C7—H7A	119.2	O9'—C35'—H35F	109.5
C6—C7—H7A	119.2	H9A—C35'—H35F	160.1
C7—C8—C9	117.1 (2)	H35D—C35'—H35F	109.5
C7—C8—H8A	121.4	H35E—C35'—H35F	109.5
C9—C8—H8A	121.4	C33—N10—C32	121.9 (3)
C8—C9—N1	130.6 (2)	C33—N10—C31	121.23 (17)
C8—C9—C4	120.7 (2)	C32—N10—C31	116.8 (2)
N1—C9—C4	108.7 (2)	N10—C31—H31A	109.5
N7—C10—C11	108.52 (18)	N10—C31—H31B	109.5
N7—C10—H10A	110.0	H31A—C31—H31B	109.5
C11—C10—H10A	110.0	N10—C31—H31C	109.5
N7—C10—H10B	110.0	H31A—C31—H31C	109.5
C11—C10—H10B	110.0	H31B—C31—H31C	109.5
H10A—C10—H10B	108.4	N10—C32—H32A	109.5
N3—C11—N4	113.1 (2)	N10—C32—H32B	109.5
N3—C11—C10	123.4 (2)	H32A—C32—H32B	109.5
N4—C11—C10	123.4 (2)	N10—C32—H32C	109.5
N4—C12—H12A	109.5	H32A—C32—H32C	109.5
N4—C12—H12B	109.5	H32B—C32—H32C	109.5
H12A—C12—H12B	109.5	O7—C33—N10	125.6 (3)
N4—C12—H12C	109.5	O7—C33—H33A	117.2
H12A—C12—H12C	109.5	N10—C33—H33A	117.2
H12B—C12—H12C	109.5	C34—O8—H8O	102.5
N4—C13—C14	131.4 (2)	O8—C34—H34A	109.5
N4—C13—C18	106.1 (2)	O8—C34—H34B	109.5
C14—C13—C18	122.5 (2)	H34A—C34—H34B	109.5
C15—C14—C13	116.5 (2)	O8—C34—H34C	109.5
C15—C14—H14A	121.7	H34A—C34—H34C	109.5
C13—C14—H14A	121.7	H34B—C34—H34C	109.5
C14—C15—C16	121.6 (2)	O3—Cl—O4	110.16 (19)
C14—C15—H15A	119.2	O3—Cl—O6	109.73 (16)
C16—C15—H15A	119.2	O4—Cl—O6	111.24 (17)
C17—C16—C15	121.3 (2)	O3—Cl—O5	108.9 (2)
C17—C16—H16A	119.3	O4—Cl—O5	108.55 (16)
C15—C16—H16A	119.3	O6—Cl—O5	108.25 (14)
C16—C17—C18	117.6 (2)		

Symmetry codes: (i) −*x*−1, −*y*−1, −*z*.

### Hydrogen-bond geometry (Å, °)

**Table:**

*D*—H···*A*	*D*—H	H···*A*	*D*···*A*	*D*—H···*A*
O8—H8O···O7^ii^	0.84	1.93	2.768 (3)	180

Symmetry codes: (ii) −*x*+1, −*y*+1, −*z*+1.

## References

[bb1] Allen, F. H., Kennard, O., Watson, D. G., Brammer, L., Orpen, A. G. & Taylor, R. (1987). *J. Chem. Soc. Perkin Trans. 2*, pp. S1–19.

[bb2] Bruker (1997). *SHELXTL* Bruker AXS Inc., Madison, Wisconsin, USA.

[bb3] Higashi, T. (1995). *ABSCOR* Rigaku Corporation, Tokyo, Japan.

[bb4] Rigaku/MSC (2004). *RAPID-AUTO* Rigaku/MSC, The Woodlands, Texas, USA.

[bb5] Sheldrick, G. M. (2008). *Acta Cryst.* A**64**, 112–122. 10.1107/S0108767307043930 18156677

[bb6] Spek, A. L. (2003). *J. Appl. Cryst.* **36**, 7–13.

[bb7] Youngme, S., Phatchimkun, J., Sukangpanya, U., Pakawatchai, C., Chaichit, N., Kongsaeree, P., Krzystek, J. & Murphy, B. (2007). *Polyhedron*, **26**, 871–882.

